# Dissimilar impact of type 2 diabetes on cardiovascular outcomes according to age categories: a nationwide population study from Hungary

**DOI:** 10.1186/s12933-018-0751-7

**Published:** 2018-07-27

**Authors:** Zoltán Kiss, György Rokszin, Zsolt Abonyi-Tóth, György Jermendy, Péter Kempler, Dániel Aradi, István Wittmann

**Affiliations:** 1 2nd Department of Medicine and Nephrological Center, Faculty of Medicine, University of Pécs, Pacsirta str. 1, Pecs, 7624 Hungary; 2 RxTarget Ltd., Szolnok, Hungary; 3 University of Veterinary Medicine, Budapest, Hungary; 4 Bajcsy-Zsilinszky Hospital, Budapest, Hungary; 5 I. Department of Medicine, Faculty of Medicine, Semmelweis University, Budapest, Hungary; 6 Heart Centre Balatonfüred and Heart and Vascular Centre, Semmelweis University, Budapest, Hungary

**Keywords:** Type 2 diabetes mellitus, Cardiovascular outcome, Cardiovascular morbidity, All-cause mortality, Myocardial infarction, Stroke

## Abstract

**Background:**

The excess risks of mortality and cardiovascular morbidity among patients with type 2 diabetes mellitus (T2DM) is well known. In this nationwide study, we assessed risks of mortality and cardiovascular events comparing patients with T2DM and matched controls.

**Methods:**

We identified patients with T2DM in a retrospective cohort study using the database of the National Health Insurance Fund between 1 January 2010 and 31 December, 2013. Controls were randomly included and matched according to age, gender, and zip code of residence. Patients were divided into subgroups according to age decades for outcome analyses.

**Results:**

During the mean follow-up period of 2.3 years, 152,678 patients with T2DM and 305,356 matched controls were included. Patients with T2DM showed significantly higher risk for all-cause mortality (HR 1.26, 95% CI 1.22–1.29, p < 0.0001), myocardial infarction (HR 1.81, 95% CI 1.69–1.94, p < 0.0001) and stroke (HR 1.40, 95% CI 1.35–1.46, p < 0.0001) compared to matched controls. The higher risk associated with T2DM for mortality, myocardial infarction and stroke differed significantly between age groups (p_interaction_ < 0.05 for all outcomes) with significantly higher risk observed in younger patients.

**Conclusions:**

The risk of cardiovascular outcomes and all-cause mortality is significantly higher in patients with T2DM. Notably, the relative hazard increases with decreasing age suggesting that younger patients with T2DM should receive more attention for cardiovascular prevention.

## Background

Cardiovascular diseases in T2DM are the leading cause of death, occurring with a span of typically 14.6 years [1, 2], and the severity and the prevalence are significantly greater than in non-diabetic population [3]. Despite significant improvement in diabetes care, several studies have painted to substantially increased mortality and cardiovascular morbidity in patients with T2DM [4]. In Ontario, Canada, and in UK, the mortality rate ratio of diabetes dropped from 1.90 to 1.51 and from 2.14 to 1.65, showing higher corresponding rate ratios in younger cohort [5].

In a recently published Swedish registry study, the risk of any cause of death was also significantly higher among patients with T2DM compared to controls (adjusted hazard ratio, HR 1.15, 95% CI 1.14–1.16). The risk of all-cause and cardiovascular death increased with younger age [6]. The Emerging Risk Factors Collaboration reported 1.80 hazard ratio, for all-cause death in T2DM patients as compared to persons without it [7]. Norhammar assessed the risk of cardiovascular morbidity in Swedish population, showing an excess risk ratio for myocardial infarction (1.68–1.74) and stroke (1.45–1.54) in the T2DM group as compared to the non-diabetic Swedish population [8]. A similar result was presented in U.S. population with a relative risk ratio for myocardial infarction 1.8 (1.3–2.3) and stroke 1.5 (1.1–2.0) [9].

Cardiovascular morbidity and mortality is one of the highest in Hungary among OECD countries. In a 2013 report, Hungary was the second behind Slovakia in case of mortality rates for CVD with 598 deaths per 100.000 populations [10].

We designed our study to explore cardiovascular morbidity and mortality in the T2DM population using data of all patients diagnosed between 2010 and 2013 and compared to a control population matched by age, gender and residence extracted from the database of the Hungarian National Health Insurance Fund. We aimed to explore age-dependent effects of T2DM on mortality, myocardial infarction and stroke in subgroups of patients defined by decades. The Hungarian National Health Insurance Fund database is a nationwide insurance system collecting patient ID-based ICD-10 code information from all in- and out-patient visits within primary and hospital care as well as from all prescription of drugs which are reimbursed in Hungary. In addition to collecting information on diagnosis, the NHIF database also records treatments and all therapeutic procedures, length of hospital stay and the in and out of hospital all-cause mortality. The NHIF covers close to 100% of Hungarian population, however there are certain (but low level) private care visits which are out of our investigation. During the investigated period, all type 2 diabetes treatments were reimbursed which allows us to identify T2DM patients.

On the other hand, NHIF database does not contain personalized information on laboratory test results e.g. HbA_1c_ or cholesterol level as well as does not give information on patients’ characteristics e.g. weight and BMI.

## Methods

### Study design and overview

In our retrospective cohort study, cases of T2DM patients (n = 152,678) starting antidiabetic therapy between 1 January, 2010 and 31 December, 2013 were retrospectively extracted from the database of the National Health Insurance Fund as an anonymized, aggregated patient data, and were considered as T2DM patients if having antidiabetic treatment (ATC A10) but not matching previously detailed and published criteria for type 1, insulin dependent diabetes and not having polycystic ovarium syndrome (ICD 10 E282) [11].

Our main goal was to investigate the risk of cardiovascular morbidity and all-cause mortality since the diagnosis of diabetes. For this reason, we needed to identify the date of diagnosis of diabetes, this way only incident population met our criteria. Onset of diabetes was defined as the first occurrences of diabetes ICD code (E10–E14) in the database for a patient or the first antidiabetic treatment, whichever occurred first. Combined records of ICD E10–14 and ATC A10 treatment (with the exclusion of PCOS diagnosis) together with specified algorithm for excluding type 1 diabetes ensures the inclusion of all T2DM patients in Hungary who are involved into national diabetes care.

The control cohort (n = 305,356) was randomly selected from the total population recorded in NHIF database by matching the age at diabetes onset, gender and zip code of residence with the T2DM cohort resulting in two controls for each DM patient, matching 1:2. In the case of controls no diabetes related ICD codes or antidiabetic treatment were recorded.

The data source included information on mortality for any causes, cardiovascular complications of diabetes like incidence of myocardial infarction (ICD-10 I21–24) and ischemic and hemorrhagic stroke (ICD-10 I61–63, G4630, G4640, G4580, G4590) in in- and out-patient records. The International Classification of Diseases (ICD) codes of 9th and 10th Revisions, were used to define acute myocardial infarction, stroke diagnoses from 1 January, 2010 onward. Dates of death were also retrieved from the National Health Insurance Fund database, however this database does not include the cause of death, therefore we used only the cases of death from any type of causes (all-cause mortality) in our analysis.

All-cause mortality and cardiovascular morbidity as myocardial infarction and stroke were assessed in a period of 58 months, from 1 January, 2010 till 31 October, 2014, by comparing T2DM with the matched control population. In both T2DM and control patients, subgroups were defined according to decades of age resulting in subgroups of > 70, 61–70, 51–60, 41–50, 31–40 and < 31. Patients were also evaluated according to gender.

Limitation of the NHIF data investigation is that allows only detection of use of reimbursed drugs. Statin therapy during the investigated period was reimbursed, but blood pressure lowering and antiplatelet therapies are not completely reimbursed in Hungary, consequently these information do not exist in the NHIF database.

This study was approved by the Regional Research Ethics Committee of the Medical Center, University of Pécs, Medical School, Hungary (Study License Number: 6962/2017) and run without commercial sponsorship. The study protocol was also reviewed and confirmed by the National Health Insurance Fund (NHIF) (Identification Number: S04/161/2016).

### Statistical analysis

Survival analyses were performed by Cox regression and age and gender matched groups were used. Our model runs separately for the sexes and the individual age groups, consequently the proportionality criterion for cox does not deteriorate if the same parentage is not the same for different gender or age groups). The final result was the adequately weighted average of the results in the different groups. This approach ensured correction for gender and age group. As the risk of death was higher in the first 4 months after the diagnosis of diabetes, and the risk of stroke and myocardial infarction was also higher in the 1st month, time dependent factor was used to separate this short-term effect from the long-term effect. Simultaneous confidence intervals including ratio of hazard ratios were calculated using contrast matrix.

Mean of age and follow-up time were compared using Welch’s two-sample test. Proportion of statin treatment, prior stroke and myocardial infarction were compared using Chi square test.

All analyses were performed by using of R Software, version 3.4.2 (2017-09-28), applying survival, survminer and multcomp packages [12].

## Results

### Study population

Between 1 January, 2010 and 31 December, 2013, 152,678 patients with T2DM and 305,356 matched controls were selected for further analyses. Baseline characteristics of T2DM patients and control subjects are presented in Table 1. Among T2DM patients, 51.89% were female. Mean age at diagnosis was 59.43, 3 years lower in males compared to female patients. Mean follow-up period of the study was 2.32 years in T2DM and 2.4 in control group.

**Table 1:** Patient characteristics at baseline

	T2DM	Control	p value
Population (n)	152,678	305,356	
Age (years)	59.43 (59.4–59.5)	59.43 (59.3–59.4)	1
Female-no. (%)	79,238 (51.9)	158,476 (51.9)	1
Age (years—95% CI)	60.89 (60.8–61.0)	60.89 (60.8–61.0)	1
Male—no. (%)	73,440 (48.1)	146,880 (48.1)	1
Age (years—95% CI)	57.9 (57.8–57.9)	57.9 (57.8–57.9)	1
Year of diagnosis (range)	2010–2013	2010–2013	
Mean follow-up (year—95% CI)	2.3 (2.3–2.3)	2.4 (2.4–2.4)	< 0.0001

### Mortality and cardiovascular events

**Fig. 1:**
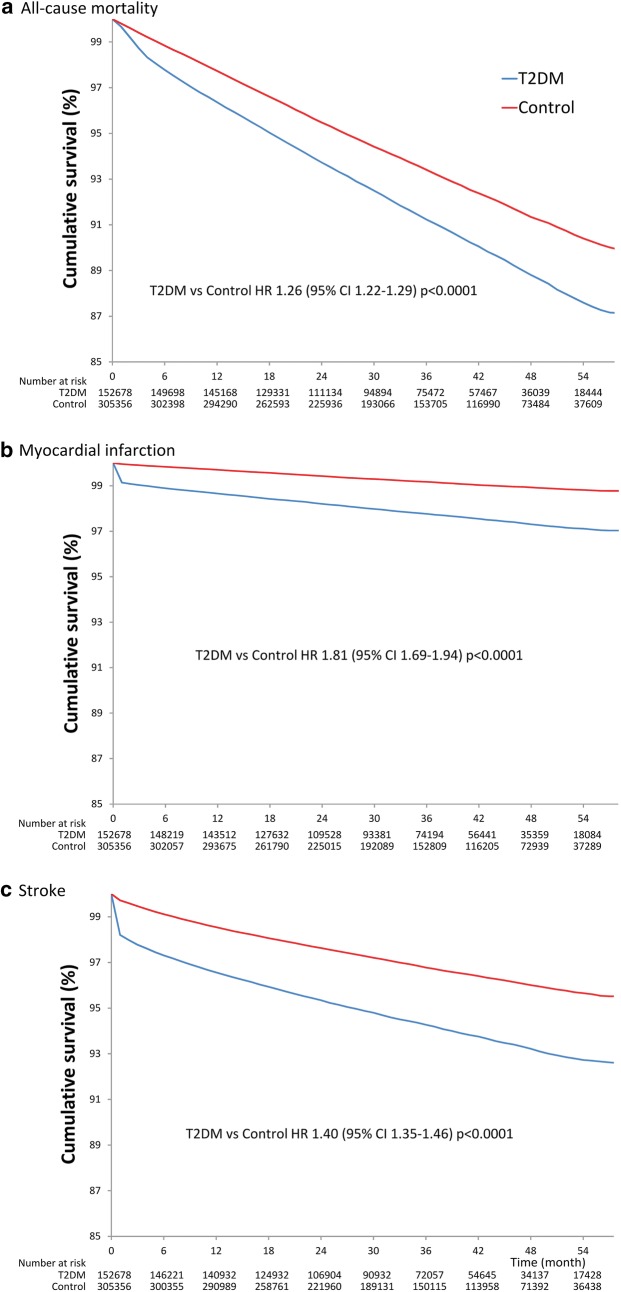
**a** Kaplan–Meier all-cause mortality free survival curve comparing T2DM with matched control; **b** Kaplan–Meier myocardial infarction free survival curve comparing T2DM with matched control; **c** Kaplan–Meier stroke free survival curve comparing T2DM with matched control

Overall, 13,413 (8.8%) of the 152,678 patients with diabetes died during the study period, as compared with 20,154 (6.6%) patients of 305,356 controls. In Cox regression analyses, hazard ratio (HR) for all-cause mortality was 1.26 (95% CI 1.22–1.29 p < 0.0001) showing a significantly higher risk for all-cause death in T2DM (Fig. 1a). Within the first 4 months of diagnosis, an even larger risk increase was found (HR for the first 4 months: 2.16, 95% CI 2.02–2.30, p < 0.0001).

Patients with T2DM showed a significantly higher risk for myocardial infarction [3330 (2.2%) vs. 2442 (0.8%) events; HR 1.81, 95% CI 1.69–1.94, p < 0.0001] (Fig. 1b) and stroke [8502 (5.6%) vs. 9475 (3.1%) events; HR 1.40, 95% CI 1.35–1.46, p < 0.0001] (Fig. 1c) compared to matched control patients. In cases of both cardiovascular complications, differences in HR-s were also significantly larger in the 1st month (HR for MI 20.19, 95% CI 16.43–24.80; p < 0.0001 and for stroke 6.52, 95% CI 5.97–7.11; p < 0.0001).

### Age-dependent analyses

**Fig. 2:**
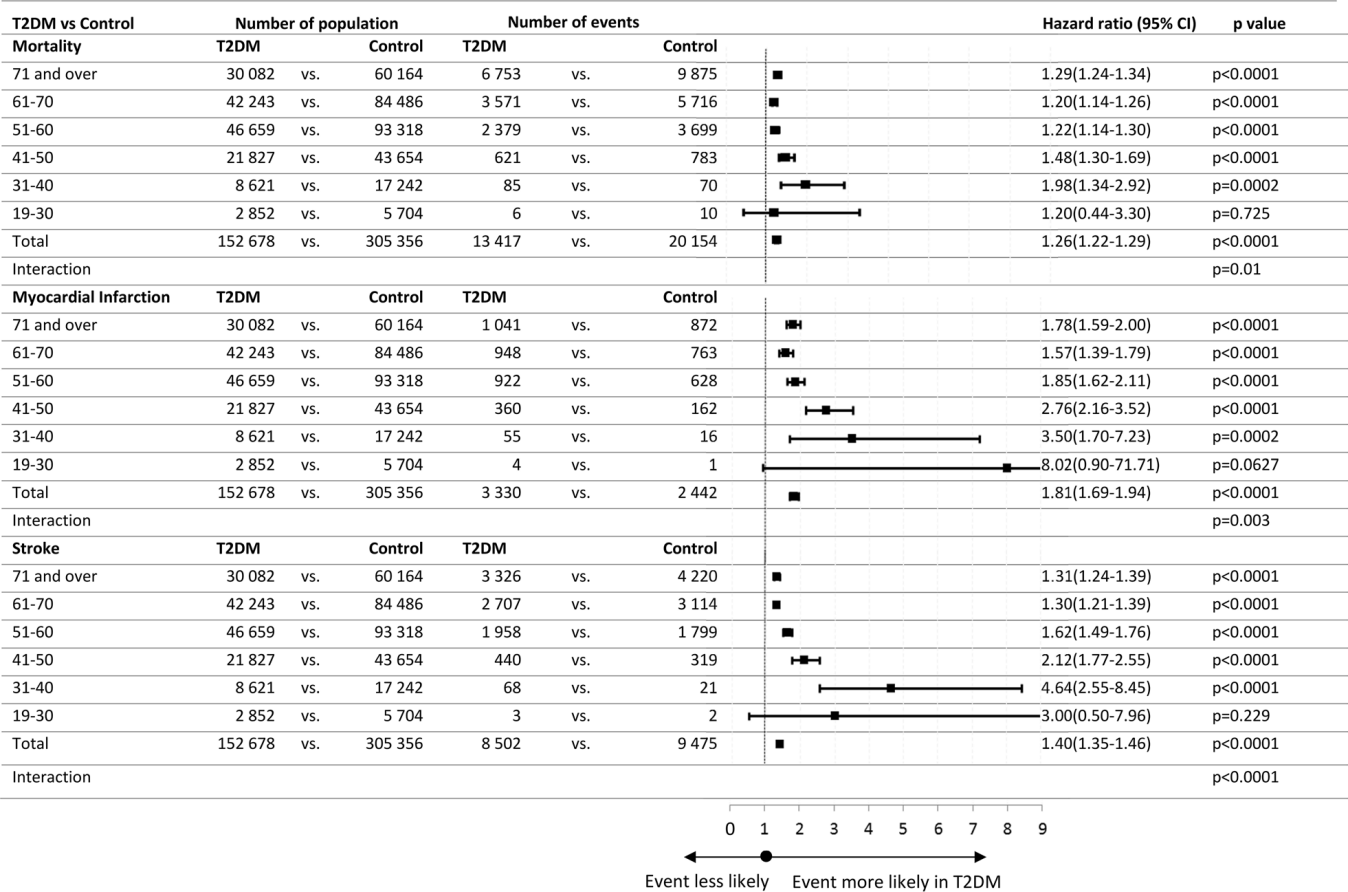
Adjusted hazard ratios for all-cause mortality, myocardial infarction and stroke according age cohorts comparing T2DM with matched control. Age-dependent interactions: mortality: p_interaction_ = 0.01, myocardial infarction: p_interaction_ = 0.003 and stroke: p_interaction_ < 0.0001

The relative risks of T2DM vs. matched controls regarding all outcomes were evaluated in pre-defined age subgroups (Fig. 2). Regarding mortality, a significant excess of risk associated with T2DM was present in all subgroups except for the smallest group of patients aged 19–30 years, presumably due to low sample size. Interaction testing showed a significant interaction of age with the risk of T2DM (p_interaction_ = 0.01). Interaction testing in age-defined subgroups for myocardial infarction and stroke provided similar findings (Fig. 2).

### Gender differences

**Fig. 3:**
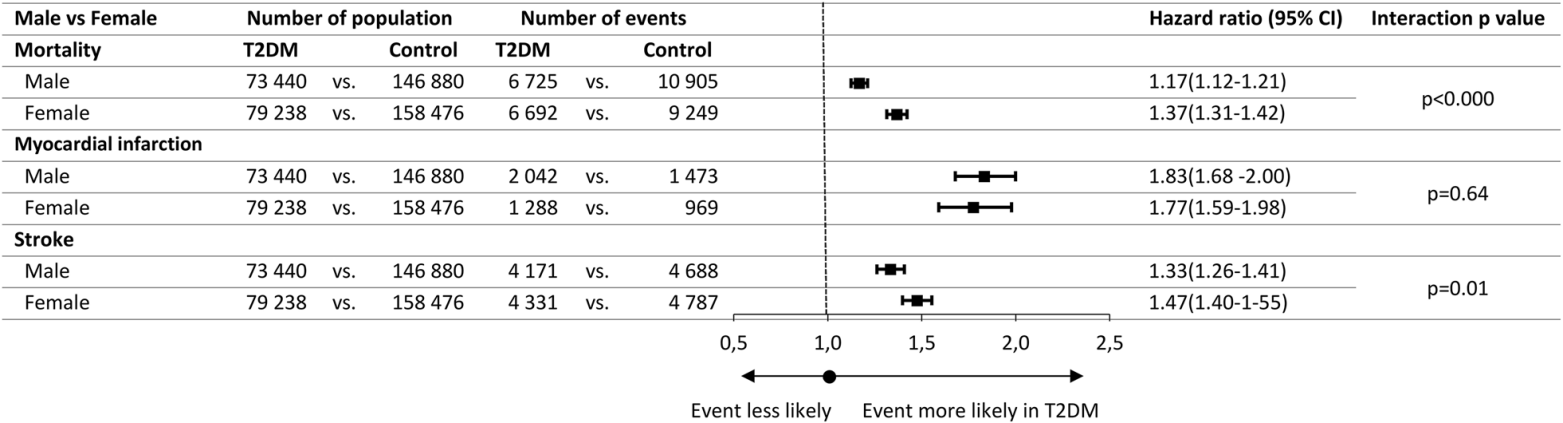
Adjusted hazard ratios for all-cause mortality, myocardial infarction and stroke according male and female within T2DM and Control population

Among patients with T2DM, 52% were male and 48% were female. Men and women with T2DM had similar mortality (9.2% vs. 8.5%), myocardial infarction (2.8% vs. 1.6%) and stroke (5.7% vs. 5.5%). In both genders, we observed a significantly higher risk for mortality (HR 1.17 and 1.37), myocardial infarction and stroke in case of T2DM compared to matched controls (Fig. 3). Based on the results of interaction testing, the risk of T2DM for mortality and stroke was larger in female patients than in male, while no gender-related difference was found in myocardial infarction (Fig. 3).

## Discussion

The main findings of the current large-scale, nationwide study can be summarized as follows:

1. Patients with T2DM have a significantly higher risk for myocardial infarction, stroke and mortality. This excess risk depends on the age of patients with significantly larger increase of relative risk observed in younger subjects than in the elderly.
2. Cardiovascular events and mortality were higher in both genders with T2DM, however; the risk of stroke and mortality was higher in female patients compared to males. Myocardial infarction did not show gender-related differences.

### Age related differences

The excess risk for cardiovascular events in patients with T2DM is well described in literature [2, 3, 13]. Similarly, prior reports suggested an age-dependent elevation of risk associated with T2DM [1]. In the Tancredi study, the authors provided a detailed analysis on the background of higher mortality rate in younger T2DM population [6]. They explained the higher mortality of younger patients by the higher rate of smoking (38% vs. 24% control) and significantly higher incidence of obesity (56% vs. 14%).

In our earlier paper, we found age-related differences in statin medication adherence in Hungary, where the younger cohort presented significantly lower, 7.9–11.8% adherence during 12 months, while rates of adherence were 15.9–23.5% in older cohorts. This feature may be one of the many that contribute to the higher cardiovascular risk profile of younger Hungarians [14], because the correlation between the use of statins and improvement of survival is well-known especially in younger age [15].

### Comparisons of mortality data

In our nationwide study, all-cause mortality was 37.89 per 10,000 person-years between 2010 and 2013, which is comparable with data presented by Tancredi from a Swedish diabetes registry [6] with 38.64 per 10,000 person-years. We found a similar incidence of mortality in the control group as well with 30.30 vs. 28.45 in Swedish versus Hungarian patients per 10,000 person-years in non-diabetic populations. Norhammar investigated the same Swedish T2DM population presenting data for period between 2010 and 2013, when the mortality ratio varied similarly between 1.29 and 1.27, versus the non-diabetic population [8]; a finding similar to our results.

### Comparisons of cardiovascular morbidity data

Notably, the highest relative risk increase was found in case of myocardial infarction (HR 1.81). Data from the Swedish registry by Norhammar related to the same time period seems to be of similar effect size for myocardial infarction to our findings (between 1.71 and 1.68) [8]. Gregg also published a comparable risk elevation for the coronary events in 2010, with relative risk of 1.8 comparing T2DM population to a non-diabetic population [9].

The hazard ratio in T2DM for stroke in Hungary was also comparable to the Swedish and US data detailed earlier. While Gregg reported a 50% risk increase in stroke in 2010, Norhammar measured hazard ratios of 1.47–1.45 between 2010 and 2013. Our nationwide survey showed a 1.40 hazard ratio. In a paper using the same NHIF database of the year 2008, Szőcs et al. reported a 43.3 incidence per 10,000 in a non-selected, non-diabetic population for stroke in Hungary [16].

Cardiovascular complications in T2DM patients have significant impact on the outcome leading to higher resource utilization in younger patients with CVD [17]. High risks for cardiovascular events in our study highlights the need of better treatment, particularly for younger diabetic patients. Achievement of therapeutic target in a secondary prevention of MI, or stroke in T2DM patients was reported to be low in an Austrian-German multicenter analysis [18], and another study confirmed these findings [19].

Several recent studies reported significant improvement in cardiovascular morbidity and mortality outcomes using new options of treatment strategies in the secondary prevention, as e.g. the EMPA-REG OUTCOME [20] and the CANVAS study [21], which proved the benefits of SGLT-2 inhibitors. However, a cardiovascular neutrality was verified in the trials using DPP4-inhibitor therapies [22, 23].

Retrospective cohort based study represented a beneficial change of risks in a robust study, where a decreased risk of cardiovascular complications was detected diabetic patients in Korean during the 2006–2013 study period [24].

Some authors hypothesize further improvement in the goal attainment and this way better achievements in the cardiovascular prevention [18], but others suggest cautiousness with potential overtreatment and its consequences [25] relating to the increased risk of hypoglycemia related cardiovascular disease [26]. However, benefit of the intensified intervention could be supposed in the prevention of cerebrovascular diseases in T2DM patients [25] and carotid and lower extremity ultrasound examination may further improve the identification of cardiovascular risk [27].

### Gender differences

When assessing mortality and cardiovascular morbidity according to genders, we found a slightly increased mortality risk for males in the T2DM population. However, if we compare the T2DM groups to the matched controls, both genders show a higher risk in T2DM than controls, with a significantly higher relative risk for stroke and mortality observed in females. This is in line with general observations, which suggest that T2DM increases the risks (especially postmenopausal) of females more than that of males [28], and treatment for women with diabetes was recommended to be more aggressive to that of men.

### Backgrounds of cardiovascular events

Though we do not have data for direct comparison, we found a relatively early diabetes onset in Hungary comparing to other Western countries, recording 59 year as a mean age at the diagnosis of T2DM (57 years for males and 60 for females). While similar cohort studies showed a diabetes onset over 65 years, such as the Swedish diabetes registry present 65 mean years between 1998 and 2011. This difference may be attributed to the higher incidence of risk factors for diabetes in Hungary. In the OECD obesity report [29], Hungary is among the top countries having the highest incidence rate for obesity. This survey reported a 30% incidence rate for our country in 2015, while Sweden was among countries at the lower end, recording 12.3% of the population reaching the criteria of obesity. Regarding obesity and related metabolic syndrome there is no additive risk for cardiovascular morbidity in case of the syndrome compared to the single components of it [30].

### Early events

The probability of myocardial infarction and stroke cases and consequently incidence of all-cause mortality was higher shortly after diabetes diagnosis. This result may come due to late diagnosis of diabetes at certain patients, which means, most of these patients missed the primary prevention systems in primary care and appeared in NHIF with diabetes after myocardial infarction or stroke and/or consequent mortality. This result highlights the need of better primary prevention care strategies in Hungary.

### Strengths and limitations

Our analysis has strengths and weaknesses, as well. The sample size of the patient group, the high number of events, and the long follow-up provided sufficient power for the analyses. The limitation of the study is that it includes patients treated by antidiabetic agents only, and not involved diabetics on life style modification. Further weakness of our trial is that we were not able to get data about hemoglobin A_1c_, cholesterol level, and cause of death as well as we could not record more detailed risk factors and we did not have option to evaluate the impact of obesity. Moreover, in our study, HR-analyses are not fully adjusted for all known cardiovascular risk factors, and with the given investigation period, we evaluated incident patients with (on average) 2.3 years of follow up, only.

## Conclusions

T2DM patients in Hungary showed a significantly higher risk for stroke, myocardial infarction and mortality. The excess risk associated with T2DM increased by decreasing age of the evaluated group of patient group suggesting an important age-dependent interaction. More efforts should be focused on the younger population to improve the prevention of cardiovascular events.

## Abbreviations

CHD: coronary heart disease
HR: hazard ratio
ICD: International Classification of Diseases
NHIF: National Health Insurance Fund
OECD: The Organisation for Economic Co-operation and Development
T2DM: type 2 diabetes mellitus
